# Correlation analysis between digital photography measurement of trunk deformity and self-image perception in patients with idiopathic scoliosis

**DOI:** 10.1186/1748-7161-9-S1-O8

**Published:** 2014-12-04

**Authors:** Antonia Matamalas, Joan Bago, Elisabetta DAgata, Ferran Pellise

**Affiliations:** 1 Hospital Vall Hebron, Barcelona, Spain

## Introduction

Trunk deformity in idiopathic scoliosis has been fully analyzed using different surface metrics but all of them are expensive and cannot be widely used. Recently it has been suggested that some measures of trunk deformity obtained in digital photography can be useful in the assessment of trunk deformity. Some asymmetry measures have been proposed but the relationship between these measures and patients’ self-image perception has not been established.

## Aim

To assess the validity of a clinical assessment tool of the trunk deformity based on photographs as compared to self-assessed appearance questionnaires.

## Study design

Cross-sectional study. Concurrent validity between postural indexes obtained from digital photographs and self-assessed appearance questionnaires.

## Methods

Front and back digital photographs of patients with idiopathic scoliosis (Cobb angle >25º) were obtained. Shoulder, armpit and waist angles in addition to trunk asymmetry indices were calculated on front and back photographs with Surgimap software. All patients completed SRS-22, SAQ and TAPS questionnaires. The Spearman´s rank correlation coefficient (r) was used to estimate concurrent validity between both methods.

## Results

80 consecutive patients (68 females), mean age 20.3 years old (range 12 to 40 years) were included. Mean Cobb angle was 45.9º (range 25.1º to 77.2º).

A moderate but significant correlation was found between waist height angle and TAPS (r=-0.34) and SAQ appearance subscale (r= 0.35). SRS22 image subscale did not correlate with any photographic measure. Shoulder height angle and trapezium angle ratio correlated significantly with SRS22 Pain (r=-0-34) and SRS22 subtotal (r=-0.23). Any other correlation between body image perception instruments and other photography measurements was found.

## Conclusion

Waist height angle measured with digital photography is moderately correlated with perceived trunk appearance. Trunk asymmetry is poorly correlated with self-assessed appearance whereas shoulder asymmetry is correlated with pain and quality of life.

